# The Role of the Gut Microbiota in the Gut–Brain Axis in Obesity: Mechanisms and Future Implications

**DOI:** 10.3390/ijms22062993

**Published:** 2021-03-15

**Authors:** Jamie van Son, Laura L. Koekkoek, Susanne E. La Fleur, Mireille J. Serlie, Max Nieuwdorp

**Affiliations:** 1 Department of Endocrinology and Metabolism, Amsterdam UMC, location AMC, University of Amsterdam, 1105 AZ Amsterdam, The Netherlands; j.vanson@amsterdamumc.nl (J.v.S.); l.l.koekkoek@amsterdamumc.nl (L.L.K.); s.e.lafleur@amsterdamumc.nl (S.E.L.F.); m.j.serlie@amsterdamumc.nl (M.J.S.); 2 Department of Vascular Medicine, Amsterdam UMC, Location AMC, University of Amsterdam, Meibergdreef 9, 1105 AZ Amsterdam, The Netherlands

**Keywords:** gut microbiota, obesity, gut–brain axis, dysbiosis, satiety

## Abstract

Interaction between the gut and the brain is essential for energy homeostasis. In obesity, this homeostasis is disrupted, leading to a positive energy balance and weight gain. Obesity is a global epidemic that affects individual health and strains the socioeconomic system. Microbial dysbiosis has long been reported in obesity and obesity-related disorders. More recent literature has focused on the interaction of the gut microbiota and its metabolites on human brain and behavior. Developing strategies that target the gut microbiota could be a future approach for the treatment of obesity. Here, we review the microbiota–gut–brain axis and possible therapeutic options.

## 1. Introduction

The gut microbiota consist of trillions of microorganisms within the gastrointestinal tract and contain over 100 times more genes than the human genome [1]. Diverse and stable microbiota is crucial for human health and essential for gut homeostasis, metabolism of nutrients, supply of microbial metabolites, and defense against pathogens [2]. Microbial dysbiosis, defined as a shift in the composition of bacteria from a diverse to a maladaptive and pathogenic profile, is associated with several pathologic conditions (e.g., obesity, type 2 diabetes mellitus, and cardiovascular disease) [3,4,5,6]. Over the last decades, the prevalence of obesity, defined as body mass index (BMI) ≥30 kg/m^2^, has dramatically increased. According to the World Health Organization, 650 million adults had obesity in 2016 [7]. Obesity occurs when energy intake exceeds energy expenditure and is a complex multifactorial condition that includes both biological and environmental factors. Obesity is a major health problem and is associated with increased morbidity and mortality [8,9] as well as high socioeconomic burden [10]. This has led to extensive research. However, currently besides bariatric surgery, successful treatment options are still limited [11].

Obesity is characterized by perturbations in homeostatic and hedonic feeding behavior [12]. During feeding, there is a complex feedback system between the gut and the brain, also known as the gut–brain axis. The importance of the gut–brain axis in energy homeostasis has long been reported [13,14]. More recent research has started to uncover the link between the gut microbiota and the central nervous system (CNS) [15]. Studies with germ-free mice have shown that the gut microbiota is essential for brain development and behavior [16,17].

Here, we aim to review the interaction between the gut microbiota and CNS in obesity and will highlight several underlying mechanisms and possible treatment strategies. 

## 2. Gut Microbiota in Obesity

In healthy adults, the gut microbiota is dominated by organisms from the Bacteroidetes and Firmicutes phyla [18]. In individuals with obesity, lower relative proportions of Bacteroidetes and higher proportions Firmicutes have been observed compared with lean controls, with a significant increase of Bacteroidetes after weight loss [3]. Additionally, comparison of twins with normal weight or obesity showed a lower bacterial diversity and Bacteroidetes proportion but higher Actinobacteria in obesity. However, there was no significant difference in Firmicutes proportion [19]. Conversely, a meta-analysis did not find a clear association between the Firmicutes/Bacteroidetes ratio and obesity, underscoring a potentially more complex system involved [20]. Nevertheless, several studies have reported that weight loss can reciprocally influence the gut microbiota composition [21,22]. In this regard, it has been suggested that in individuals with obesity, the gut microbiota may be more efficient in harvesting energy, leading to more fat storage compared with lean individuals [23,24]. Indeed, research in mice revealed that colonization of germ-free mice with microbiota of conventionally raised mice significantly increased body fat despite reduced food intake [23]. Moreover, the gut microbiota from human or mice donors with obesity resulted in greater increase in body fat than the microbiota from lean donors in mice [24,25]. However, findings are inconsistent, and it is not clear if and how this will translate to humans [26]. 

Obesity is characterized by the presence of low-grade inflammation, which increases the risk for comorbidities, such as insulin resistance, cardiovascular disease, and cancer [27,28]. The precise mechanisms behind this inflammatory state are still largely unknown, but several factors have been implicated, including the gut microbiota. It is thought that changes in gut microbiota composition in obesity lead to increased gut permeability [29]. Disruption of this intestinal barrier leads to passage of components, such as lipopolysaccharide (LPS), an endotoxin produced by the lysis of Gram-negative bacteria, into the systemic circulation. These elevated plasma levels of LPS can initiate inflammatory cascades in adipose tissue, leading to the production of proinflammatory cytokines [30], and can also induce neuroinflammation [31]. It has indeed been shown that humans with obesity have significantly higher plasma LPS levels after a high-fat meal compared with lean controls [32], further underscoring the potential contributing role of the gut microbiota in the low-grade inflammatory state in human obesity. 

## 3. The Microbiota–Gut–Brain Axis

The gut–brain axis is a complex bidirectional pathway that is essential for metabolic homeostasis. The gut can interact with the CNS to transfer information on nutritional status through a variety of mechanisms, including enteroendocrine cells (EECs), the vagus nerve (VN), and the enteric nervous system (ENS). Microbial-produced metabolites can modulate these signals. In this section, we will provide a brief overview of the microbiota–gut–brain axis.

During feeding, several neuroendocrine mechanisms are activated. EECs are located throughout the epithelium of the gut and respond to nutrient and mechanical stimuli by secreting hormones and neurotransmitters that have a key role in metabolism, including serotonin (5-hydroxytryptamine), ghrelin, cholecystokinin (CCK), peptide YY (PYY), and glucagon-like peptide 1 (GLP-1) [33,34]. These endocrine hormones mediate their effect on secretion (insulin, gastric acid, and bile acids), gut motility, and food intake through vagal afferent neurons or ENS or through release into the bloodstream [35,36,37]. Indeed, gut-microbiota-derived metabolites (e.g., short-chain fatty acids (SCFAs)) can modify the release of these hormones and neurotransmitters [38,39]. 

Beyond hormonal signaling, neuronal orchestration of the gut–brain axis is gaining more and more attention. In this regard, the VN is the longest nerve in the human body and plays a key role in gut–brain signaling [40]. It is a mixed nerve containing approximately 80% afferent and 20% efferent fibers that transmit information from the viscera to the brain and vice versa [41]. Signals from the VN are integrated in the nucleus of the solitary tract (NTS), which then projects signals to areas important for energy balance, such as the hypothalamic arcuate nucleus (ARC), the parabrachial nucleus, and the dorsal motor nucleus [42,43,44]. The VN is involved in food intake and gastrointestinal motility and has anti-inflammatory properties [45]. Its afferent fibers can be activated by gastrointestinal tract (GIT) distension, hormones, and peptides released from the EECs or microbial metabolites [46]. The VN can also be directly inhibited by ghrelin and PYY or stimulated by CCK [47,48,49]. Several studies have shown an association between microbial dysbiosis in obesity and VN innervation and signaling [50,51]. Moreover, older studies have suggested that (gastric) vagotomy in humans affects body weight, thus underscoring the driving role of VN in human metabolism and food intake [52], a finding that more recently has been endorsed in mice [53]. 

Connected to the VN is the enteric nervous system, which comprises a neural network located throughout the GIT, which controls gut motility, blood flow, and secretion. It communicates with the CNS (e.g., via the VN) but is also capable of detecting and integrating information and functioning independently [54,55]. For example, animal studies have shown that with a severed vagus nerve, the ENS continues to function [56]. The gut microbiota may signal the ENS directly through microbial-produced neurotransmitters and SCFAs or indirectly through the effect of bacterial metabolites on the EECs. Studies in germ-free mice have shown that the gut microbiota is involved in the maturation of the ENS, and also during adult age, ENS might be sensitive to microbial intervention [57,58,59].

Moreover, the dietary nutrients that are converted into plasma metabolites by the gut microbiota include short-chain fatty acids (SCFAs lactate, butyrate, propionate, acetate, and succinate), as well as gamma-aminobutyric acid (GABA), dopamine, and serotonin, which all have important functions in the gut–brain axis. Specifically, the SCFAs that are derived from dietary fibers are essential signaling molecules by binding to G protein-coupled receptors (GPR), GPR43, and GPR41 [60]. They have a role in satiety, may increase energy expenditure [61], and have also shown to have a possible role in CNS inflammation [62]. Additionally, butyrate is an important energy source for the colonocytes, but also has a strong epigenetic effect (via methylation as HDAC inhibitor) [63]. Although the majority of intestinally produced SCFAs are consumed in the intestine, a small part can stimulate the secretion of PYY and GLP-1 through interaction with EECs [64,65], thus indirectly affecting satiety. Additionally, SCFAs can suppress food intake by stimulating the VN [66] and centrally after passing the blood–brain barrier (BBB) through the circulatory system. A study in mice showed that acetate can cross the BBB and induce anorexigenic signals in the hypothalamus [67]. Besides modulating satiety, studies in obese mice also found that acetate and butyrate can increase thermogenesis and energy expenditure [61,68]. Additionally, a study in mice suggested that SCFA-mediated GPR43 activation inhibits fat accumulation in adipose tissue and promotes metabolism of glucose in the liver and muscle [69], and propionate and butyrate have been demonstrated to induce intestinal gluconeogenesis in rats [70]. In this regard, research providing insight into the role of SCFAs in obesity has been performed. For example, a study with dietary supplementation of SCFAs in mice prevented high-fat-diet-induced weight gain [71]. In humans, individuals with obesity have higher fecal concentrations of SCFAs compared with lean individuals [72,73]. However, the relevance of fecal concentrations is debatable as it does not directly correlate with circulating SCFAs [74]. Several human trials have suggested a positive effect of SCFAs on energy expenditure, glucose metabolism, and appetite [75,76,77]. Furthermore, ex vivo treatment of human adipose tissue biopsies with propionic acid increased the expression of the anorexigenic hormone leptin and reduced inflammatory factors [78,79]. Most studies in humans are associative, and our group recently showed that oral administration of SCFA butyrate does not improve glucose metabolism or vagal tone in metabolic syndrome subjects [76,80]. Additional human trials are needed to further identify the possible role of other SCFAs as intervention in obesity and obesity-related metabolic disorders. 

**Figure 1:**
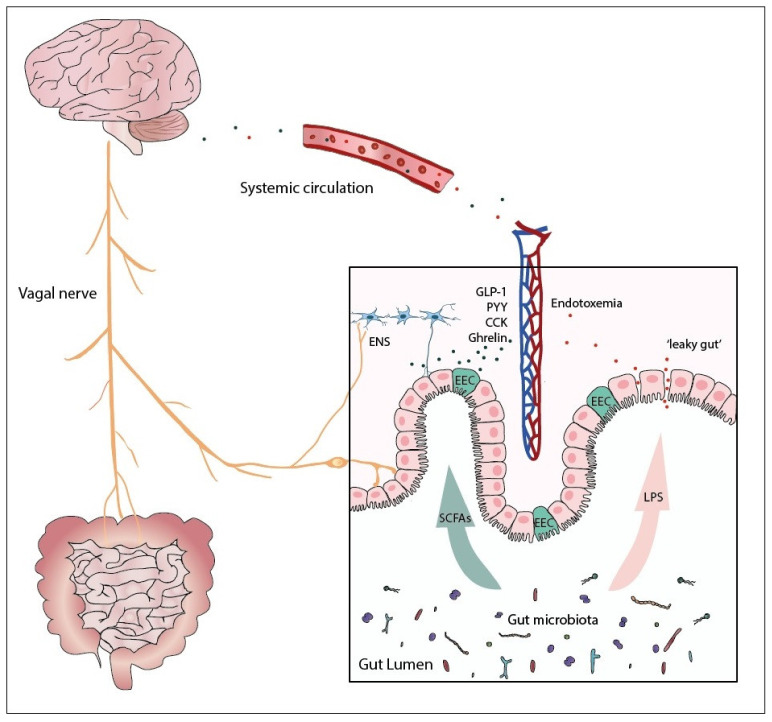
Microbiota–gut–brain axis. The gut–brain axis is a bidirectional pathway that integrates signals from the VN, hormones (e.g., GLP-1, PYY, CCK, ghrelin) secreted from the EECs and signals from the ENS. The microbiota and its metabolites, such as SCFAs, can influence the gut–brain axis through modulating these pathways. Gut permeability due to altered gut microbial composition in obesity leads to LPS leakage and endotoxemia, which in turn can induce peripheral and neuroinflammation. GLP-1, glucagon-like peptide 1; PYY, peptide YY; CCK, cholecystokinin; SCFAs, short-chain fatty acids; LPS, lipopolysaccharide; EEC, enteroendocrine cell; ENS, enteric nervous system.

In summary, there is a complex interaction between the gut and the CNS to maintain energy homeostasis, which can be modulated by the microbiota and its metabolites (Figure 1).

## 4. Gut Microbiota and Metabolites and Feeding Behavior 

The consumption of food is essential for survival. Signals of hunger and satiety motivate behavior to obtain an adequate intake of nutrients. In vertebrates, food intake is regulated by two pathways: the homeostatic and hedonic pathways. The homeostatic pathway controls energy balance and energy metabolism by mediators such as leptin and ghrelin, while the hedonic pathway is involved in the motivational aspects of food (i.e., pleasure and reward) [81]. Even though these are separate pathways, interaction between the two as well as with the gut microbiota has been reported [82]. 

### 4.1. Homeostatic Pathway

Homeostatic control of feeding is focused on energy homeostasis. Before, during, or after ingesting a meal, neural, substrate-driven, and hormonal pathways signal the brain on the nutrient status. The ARC plays a central role in food intake pathways and receives input from circulating metabolic substrates that pass the BBB and through vagal afferents, which activate the NTS, inducing a signal to the ARC [83,84]. There are two major neuronal populations that play an important role in food intake and energy balance. One group consists of the orexigenic GABAergic neurons that express neuropeptide Y (NPY) and agouti-related protein (AgRP), which during acute activation increase food intake and reduce energy expenditure [85]. The second group consists of glutamatergic anorexigenic neurons that express pro-opiomelanocortin (POMC) and cocaine- and amphetamine-regulated transcript (CART) [86]. Chronic POMC neuron activation suppresses feeding behavior. Activation of POMC neurons leads to alpha-melanocyte-stimulating hormone (α-MSH) release, which then activates melanocortin-4-receptor (MC4R) and the melanocortin satiety pathway [87]. AgRP is an inverse agonist of the MC4R [88]. 

With regard to hormonal regulation of the homeostatic pathway ghrelin, an orexigenic hormone, mainly produced in the stomach by the EECs, rises before meals and is suppressed after food ingestion [89,90]. Ghrelin increases gastric motility and gastric acid secretion and simulates food intake, but also is involved in glucose metabolism, learning and memory, taste, and reward [91]. Moreover, ghrelin passes the BBB and activates NPY/AgRP neurons. In addition, ghrelin mediates GABA release from the NPY/AgRP neurons, leading to inhibition of POMC/CART. The effect of ghrelin on reward-driven feeding has more recently been described [92,93]. In obese individuals, plasma ghrelin concentrations are lower than in lean controls, suggesting that these plasma concentrations do not contribute to the increased food intake [94]. In line with this, leptin, an anorexigenic hormone, is produced by white adipose tissue, and circulating plasma concentrations are related to body fat mass [95]. Leptin is a marker for long-term energy balance and signals the hypothalamus after crossing the BBB [96]. A decrease in plasma leptin concentrations has also been described as a hunger signal [97,98]. Leptin inhibits hunger signals by binding to leptin receptors in the ARC, leading to activation of POMC/CART neurons and inhibition of NPY/AgRP neurons [99]. Plasma leptin concentrations are higher in individuals with obesity compared with lean controls [95]. However, this does not lead to reduced food intake, which suggests that there is resistance to the effects of leptin. This is in line with studies with exogenous administration of leptin in obesity that have failed to show weight loss [100]. Possible mechanisms, such as reduced transport across the BBB, have been hypothesized [101]. However, leptin resistance has been observed with intact leptin transport across the BBB [102]. Impaired hypothalamic signaling has been implied to underlie leptin resistance in obesity [103,104]. Therefore, although leptin is a key signaling factor during starvation, its precise role in overfeeding is still unclear [105]. 

Although the involved gut microbiota are not known, previous studies have shown that specific bacterial strains are associated with intestinal hormone concentrations. For example, quantities of *Bifidobacterium* and *Lactobacillus* negatively correlated with ghrelin concentrations and positively correlated with plasma leptin concentrations [106]. Furthermore, in *Helicobacter pylori*-infected patients who underwent eradication therapy, an increase in the Bacteroidetes/Firmicutes ratio significantly correlated with a decrease in ghrelin concentrations, suggesting that changes in the microbiota composition could affect plasma ghrelin concentrations [107]. Additionally, a recent study showed that SCFAs were able to attenuate ghrelin-mediated signaling through the growth hormone secretagogue receptor-1a [108]. In this regard, in mice SCFAs were found to stimulate leptin production in cultured adipocyte cells through the activation of GPR41 and also increased circulating leptin concentrations after oral administration [109]. Germ-free mice have shown to have higher leptin sensitivity, resulting in greater weight loss after leptin injection, compared with conventionally raised mice on a chow diet [110], and hypothalamic expression of suppressor of cytokine signaling 3, which is associated with leptin resistance, was higher in the conventionally raised mice [110]. A recent study found that supplementation with *Lactobacillus rhamnosus* GG for 10 weeks decreased the proportion of Proteobacteria and restored exogenous leptin responsiveness in high-fat-diet mice [111].

Besides orexigenic hormonal gut–brain circuits, the previously mentioned anorexigenic peptides PYY, CCK, and GLP-1 (mainly produced by the intestinal EECs and postprandially released) also play an important regulatory role. After a meal, an increase of plasma concentrations of PYY inhibits NPY/AgRP by binding to the neuropeptide Y receptor type 2 (Y2R). This removes their inhibition on the POMC neurons, which reduces food intake [112,113]. GLP-1 plays an important role in glucose regulation by enhancing glucose-induced insulin secretion and lowering glucagon secretion, and therefore, GLP-1 receptor agonists (GLP-1RAs) have been developed as a treatment for type 2 diabetes [114]. GLP-1RAs have been shown to also induce weight loss by the reduction of food intake and gastric emptying [115,116]. In rodents, GLP-1RAs exert this effect by activating POMC/CART neurons and indirectly inhibiting NPY/AgRP through GABAergic transmission [117]. Ongoing studies are currently investigating the possibility of weight management with GLP-1RAs [118], and a recent randomized trial has reported a significant reduction in weight after treatment with the GLP-1 analogue semaglutide once weekly for 68 weeks [119]. Furthermore, GLP-1 exerts its effect on satiety and gastric emptying through GLP-1 receptors in the vagal nerve [120]. 

Moreover, CCK secretion is regulated by GLP-1, and CCK is involved in digestion by stimulating gallbladder contraction, pancreatic secretion, delaying gastric emptying and increasing satiety [121]. It was the first gut hormone that was shown to induce satiety and decrease food intake in humans [49,122]. CCK reduces food intake through activation of the vagus nerve after binding the vagal CCK-A receptors [123,124]. However, in humans, stimulation with a CCK-A receptor agonist did not result in weight loss [125], thus limiting its therapeutic benefit. A recent study in mice showed that colonization with dysbiotic microbiota led to decreased vagal innervation at the NTS, which was associated with loss of CCK-induced satiety [126]. To our knowledge, currently, little is known about the possible effects of the gut microbiota on the regulation of CCK release. However, humans with obesity who underwent Roux-en-Y gastric bypass surgery had no changes in plasma CCK even though their microbial composition significantly changed [127]. 

In this regard, there is more evidence suggesting that the next-generation (gut microbiota produced) intestinally derived metabolites do play a larger role in the regulation of host appetite and metabolism [128]. For example, caseinolytic protease B produced by *Escherichia coli* is an antigen-mimetic of α-MSH [129]. As described above, α-MSH has a central role, but local signaling in the gut has also been suggested after a study showed stimulation of the MC4R in the gut EECs induced PYY and GLP-1 release [130]. Another study found that in mice, α-MSH was upregulated after nutrient-induced *E. coli* growth and led to increased plasma PYY and GLP-1 release and activated POMC neurons [131]. Finally, imidazole propionate (ImP, derived from gut-microbiota-processed dietary histidine) results in insulin resistance and subsequent type 2 diabetes [132,133]. Moreover, recent data showed that ImP is present in the forebrains of mice, thus underscoring that it can pass the BBB and therefore affect neurodevelopmental trajectories, including the gut–brain axis [134,135]. Moreover, histidine, the precursor to ImP, has been suggested to affect GLP-1 secretion [136]. Taken together, the homeostatic pathway of feeding is a complex interaction between the periphery and the CNS. Although more research is needed, there is accumulating evidence of an interplay with the gut microbiota and the secretion and function of endogenous (incretin) hormonal potentiation. 

### 4.2. Hedonic Pathway and Microbiota

An abnormal hedonic drive and enhanced motivation, both elements of the reward system, have also been shown to play a major role in obesity [137,138,139]. The mesolimbic dopamine system centered in the striatum modulates reward and has close connection with the homeostatic system [12]. Food intake stimulates dopamine release, and the rewarding sensation can promote feeding behavior [140]. Again, leptin and ghrelin have both been identified to affect mesolimbic dopaminergic activity [93,141]. Moreover, lower binding potential of striatal dopamine D2 receptors (D2R) in humans with obesity compared with lean controls has been reported in both animal studies and single photon emission computed tomography (SPECT) studies in humans [142,143]. Additionally, it was found that individuals with obesity who were presented pictures of high-calorie food showed increased activation of regions involved in reward and motivation [144] but had less activation from actual food consumption [145]. It has been hypothesized that decreased dopaminergic signaling leads to overconsumption to compensate for lower reward feedback during feeding [146,147]. However, there is also evidence showing hyper-responsivity of reward regions, thus increasing the risk for overeating, which suggests that the response to food cues in obesity is a dynamic model that shifts over time [148].

Dopamine is involved in neurological processes, including cognition, motor function, learning, and reward, and also regulates various peripheral functions, such as insulin release [149]. In the gastrointestinal tract, dopamine reduces gut motility, modulates mucosal blood flow, and stimulates exocrine secretions [150]. A substantial portion of peripheral circulating dopamine is produced in the gastrointestinal tract [151]. Altered gut microbiota composition has been reported in several central disorders with dysregulated dopaminergic transmission, such as anxiety, depression, and Parkinson’s disease [152,153]. It is unknown whether peripherally produced dopamine has direct effects on dopaminergic pathways in the brain. It has been hypothesized that the proinflammatory state in microbial dysbiosis could contribute to these disorders [31,152]. However, evidence on a causal effect in humans is scarce.

**Figure 2:**
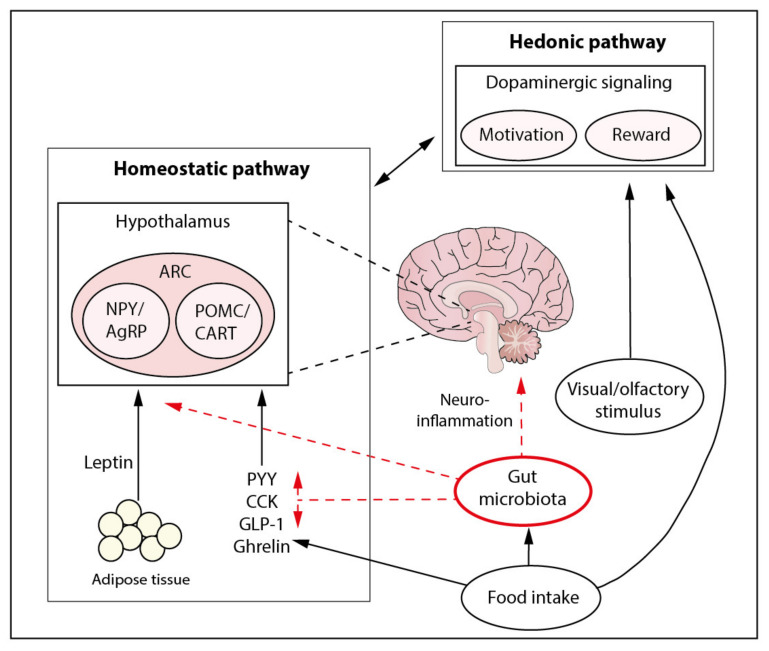
Possible mechanisms through which the gut microbiota could affect the homeostatic and hedonic pathways. The homeostatic pathway includes the processing of signals from white adipose tissue-derived leptin and GIT-produced peptides, PYY, CCK, GLP-1, and ghrelin, directly or through the VN. The hedonic pathway, which includes motivation- and reward-driven behavior through dopaminergic signaling, can be stimulated by food intake and visual and olfactory stimuli. Although the gut microbiota’s role in these pathways is still largely uncertain, several mechanisms have been hypothesized, indicated by the red dotted arrows. Altered gut microbiota composition could affect plasma leptin concentrations and sensitivity and alter the release of peptides. Additionally, microbial dysbiosis can lead to neuroinflammation, which could possibly alter both the homeostatic pathway and central dopaminergic signaling. GIT, gastrointestinal tract; ARC, arcuate nucleus; NPY, neuropeptide Y; AgRP, agouti-related protein; POMC, pro-opiomelanocortin; CART, cocaine- and amphetamine-regulated transcript; PYY, peptide YY; CCK, cholecystokinin; GLP-1, glucagon-like peptide-1.

An overview of the homeostatic and hedonic pathways and possible ways of interaction from the gut microbiota is summarized in Figure 2.

## 5. Other Neurotransmitters

Besides dopamine, the gut bacteria can modulate and produce several neurotransmitters, including GABA and serotonin. In this section, we will briefly summarize their function and how gut microbiota composition may affect them. 

### 5.1. Serotonin

Serotonin has a key role both in the brain and peripherally, including modulating satiety, anxiety, and mood and stimulating peristalsis, secretion, and vasodilation, respectively [154,155,156]. There is also evidence suggesting the role of serotonin in the regulation of glucose and lipid metabolism [157,158]. Serotonin cannot pass the BBB; thus production from tryptophan takes place separately within the periphery and the CNS. Tryptophan is an essential amino acid obtained from dietary proteins and is a precursor to several metabolites, including serotonin [159]. It is well established that the majority of peripheral serotonin is produced in the enterochromaffin cells in the (small) intestinal EECs [156,160]. Within the hypothalamus, serotonin mediates its food intake suppressant effects through inhibition of NPY/AgRP and activation of POMC neurons. However, the complete interaction between serotonin and satiety signals is not yet fully understood [155]. The serotonin 2C receptor agonist, lorcaserin, was found to significantly improve weight loss in individuals with obesity [161], but was recently withdrawn from the market after reports of increased cancer risk [162]. In obesity, evidence of decreased central serotonin signaling has been reported [147]. 

In the periphery, increased circulating plasma serotonin concentrations have been found in individuals with obesity [163]. Crane et al. found that inhibition of peripheral serotonin synthesis enhanced thermogenesis in adipose tissue, causing increased energy expenditure and protection against high-fat-diet-induced obesity in mice [164]. Gut microbes have a regulatory effect on the peripheral concentrations of serotonin [165]. The role of serotonin in obesity is complex, and the role the microbiota have on central serotonin is still unclear. However, our recent pilot study using SPECT imaging in humans suggested that the gut microbiota can directly or indirectly affect brain serotonin (and dopamine) transporter binding potential in humans with obesity via sympathetic tone [80].

### 5.2. GABA

GABA is the main inhibitory neurotransmitter. GABA is produced from its counter glutamate, which functions as an excitatory neurotransmitter. GABA has been implicated in the hypothalamic control of food intake [166,167]. Peripheral GABA production can be stimulated by several bacteria of the microbiota, such as *Bifidobacterium* and *Lactobacillus* [168]. Animal studies have indicated that treatment modulating the microbiota might improve metabolic health and behavior. For example, treatment with *Lactobacillus rhamnosus* induced alterations in GABA mRNA in the brain and reduced anxiety- and depression-related behavior via the regulation of vagal nerve tone [169], and obese mice that received the GABA-producing *Lactobacillus brevis* improved metabolically and had less depressive-like behavior [170]. Finally, we recently showed that in humans with obesity treated with fecal microbiota transplantation (FMT) from lean donors, plasma GABA concentrations increased [171].

## 6. Therapeutic Options for Modulation of the Gut–Brain Axis via Microbial Communities

Whereas gut microbiota dysbiosis seems to play a role in obesity, possibly affecting fat storage and inflammation and modulating the gut–brain axis, we now aim to discuss novel therapeutic options.

Diet plays an important role in the composition and diversity of the gut microbiome and has been identified to affect colonization, maturation, and microbiome diversity [172]. A population-based study revealed significant associations between diet and gut microbiota variation [173]. Especially Western-style diets (characterized by high intake of saturated fats and sucrose and low amounts of fiber) have been associated with metabolic disease and obesity [174] and can lead to shifts in the microbiota composition [175]. For example, high-fat diets have shown to alter the gut microbiota and increase intestinal pathogens in both animal and human studies [176,177]. However, adverse diet-related effects are likely to be person specific as subjects with higher baseline microbial diversity seemed to be more resistant to adverse microbial changes induced by high-fat [178] and sugar-containing diets [179]. In contrast, dietary nondigestible fibers (responsible for SCFA production) promote intestinal health. A meta-analysis found that a dietary fiber intervention resulted in higher levels of *Bifidobacterium* spp. and *Lactobacillus* spp., but did not affect SCFA concentrations [180]. Future studies are required to improve our understanding of the underlying mechanisms of how specific dietary components affect the gut microbiota composition and subsequent health consequences. 

In this regard, interventions with probiotics and prebiotics and FMT are discussed.

### 6.1. Probiotics

Probiotics are defined as “live microorganisms that confer a health benefit on the host when administered in adequate amounts” [181]. Intervention with probiotics might be an option for the treatment of obesity and obesity-related disorders, although it is a challenge to get viable bacterial strains into the gut because of the low pH of the stomach. Nevertheless, studies in animals and humans have shown that probiotics are associated with weight change [182,183]. Even though study results are not always consistent, a large meta-analysis showed that probiotic intervention was associated with a reduction in body weight in subjects with obesity [184]. The most commonly used probiotics are *Bifidobacterium* and *Lactobacillus* genera; however, their effect on obesity seems to be species and strain specific [183]. In line with this, oral administration with probiotics for 4 weeks was associated with affected midbrain connectivity assessed with functional magnetic resonance imaging in healthy female subjects [185]. More recently, *Akkermansia muciniphila*, a strain that has been negatively associated with obesity [186], was tested in a randomized placebo-controlled study. Although it was well tolerated and improved insulin sensitivity, there was no effect on fasting glucose or body weight when compared with placebo. Additionally, this study did not investigate aspects of the gut–brain axis [187]. All in all, it is too early to conclude that probiotics can help to improve metabolism and body weight in humans.

### 6.2. Prebiotics

Prebiotics are defined as “selectively fermented ingredients that result in specific changes in the composition and/or activity of the gastrointestinal microbiota, thus conferring benefits upon host health” [181]. Prebiotics are present in plants used for food or can be industrially produced and may be considered a future target against obesity [188]. Treatment with prebiotics in human trials led to a reduction of metabolic endotoxemia [189,190] and increased SCFA production [191] in individuals with obesity. The prebiotics inulin-type fructans have been shown to increase the growth of the beneficial lactobacilli and bifidobacteria and increase (postprandial) GLP-1 and PYY response with a concomitant decreased serum ghrelin concentration, which could affect food intake [192,193]. However, human trials studying the effect of prebiotics on weight loss have had contradictory results [194,195]. Even though accumulating data are becoming available, the precise mechanisms between prebiotics and obesity are still unclear. 

### 6.3. Fecal Microbiota Transplantation

Altering the gut microbiota through FMT has increasingly gained interest as a possible treatment for chronic diseases. During FMT, the stool of a heathy donor is infused into the patient’s intestinal tract. FMT has been successfully used as treatment for recurrent *Clostridium difficile* infections [196]. More recent studies found a possible role of fresh donor FMT in the treatment of peripheral insulin resistance [171], with a specific important role in the metabolic status of the FMT donor [197]. The transplantation of fecal microbiota from twins discordant for obesity into germ-free mice led to increased body and fat mass if the donor was obese [25]. In contrast, two placebo-controlled pilot studies in humans with obesity found that treatment with FMT capsules from lean donors did not result in significant weight loss [198] or improve metabolic health [199]. Likewise, a randomized placebo-controlled trial in adolescents with obesity did not find an effect of FMT on weight loss [200]. However, exploratory post hoc analyses did show a significant decrease in subjects with metabolic syndrome and low baseline microbiota diversity [201]. As these two studies used a different route of administering donor feces (frozen capsules instead of duodenal infusion of fresh feces potentially losing beneficial strains) and did not assess the metabolic status of the FMT donor, future studies will have to shed light on the efficacy of donor FMT on human metabolism and the CNS.

## 7. Conclusions

Currently, there are limited effective treatments available for obesity. There is accumulating evidence of the role of the gut microbiota in obesity and its interaction with the gut–brain axis. Manipulation of the gut microbiota might be a novel therapeutic option in treating obesity. However, mechanisms linking the gut microbiota and the CNS in obesity are complex. Better understanding of these mechanisms could lead to the development of microbiota-targeted therapies, such as probiotics, prebiotics, and FMT. 

## Data Availability

No new data were created or analyzed in this study. Data sharing is not applicable to this article.
